# Single‐Cell RNA Sequencing Reveals Disrupted Stromal–Epithelial Crosstalk Impairs Gut Barrier in Depression

**DOI:** 10.1002/brb3.71627

**Published:** 2026-07-28

**Authors:** Xiao‐Song Xu, Wan‐Ning Zhang, Xu‐Rui Hao, Xiao‐Yu Liu, Hong‐Chen Yu, Cai‐Xia Jia, Jian‐Ming Jiang, De‐Zhi Kong, Yan‐Ru Cai

**Affiliations:** ^1^ Research Center Hebei Provincial Hospital of Chinese Medicine Shijiazhuang China; ^2^ Graduate School Hebei University of Chinese Medicine Shijiazhuang China; ^3^ Institute of Integrated Traditional Chinese and Western Medicine Hebei Medical University Shijiazhuang China

**Keywords:** cell‒cell communication, chronic unpredictable mild stress, depressive disorder, epithelial cells, single‐cell RNA sequencing, stromal cells

## Abstract

**Background:**

Depressive disorder is commonly associated with gastrointestinal dysfunction, but the specific cellular and molecular alterations within the colonic mucosa remain poorly understood. The role of stromal–epithelial crosstalk in the colonic niche under depressive conditions is particularly unclear.

**Methods:**

A chronic unpredictable mild stress (CUMS) mouse model of depression was established and validated behaviorally. Single‐cell RNA sequencing (scRNA‐seq) of colon tissues from CUMS and control mice was performed to define cellular composition and transcriptional changes. Pseudotime trajectory, Gene Set Variation Analysis (GSVA), and cell–cell interaction analyses were used to investigate differentiation pathways and functional states. Key findings were validated using intestinal organoid cultures and immunofluorescence staining.

**Results:**

The CUMS model successfully induced depression—like behaviors. scRNA‐seq revealed a global reduction in colonic epithelial cells and a severely disrupted differentiation trajectory in depressed mice. The continuum from stem to terminally differentiated states was broken, with concomitant downregulation of the stem cell marker Lgr5 and key tight junction genes *Ocln*, *Tjp1*(encoding zonula occludens‐1, ZO‐1), and *Cldn2*. We identified a profound stromal remodeling, characterized by expansion of specific subsets: cCF1 (colonic crypt‐bottom fibroblast 1, which support the stem cell niche) and CTF (crypt‐top fibroblasts, which promote epithelial differentiation). CTF cells adopted a pro‐inflammatory, oxidative stress phenotype, while cCF1 cells exhibited a pro‐fibrotic signature involving enhanced TGF‐β and suppressed Wnt/β‐catenin signaling. Cell communication analysis demonstrated a globally enhanced interaction network between epithelial stem cells and the expanded cCF1/CTF subsets in depression. Functionally, intestinal organoids derived from depressed mice exhibited altered growth and regenerative capacity, as evidenced by reduced Ki67‐positive proliferating cells and Lgr5‐positive stem cells in immunofluorescence staining.

**Conclusion:**

This study provides a single‐cell transcriptomic atlas of the colon in depression, preliminary suggesting that disease pathology may involve functional reprogramming of the stromal niche. Expansion of disease‐specific stromal populations cCF1 and CTF may disrupt epithelial homeostasis, suggesting a potential role in impaired stem cell function, dysregulated cellular differentiation, and compromised intestinal barrier integrity. These findings suggest a potential cellular mechanism for depression‐associated gastrointestinal comorbidity and may provide a reference for the development of future treatment strategies.

## Graphical abstract

scRNA‐seq atlas of the depressed mouse colon reveals that expansion of disease‐specific cCF1 and CTF stromal subsets disrupts epithelial homeostasis via enhanced TGF‐ß and suppressed WNT/ß‐catenin signaling, impairing stem cell function, differentiation, and intestinal barrier integrity.

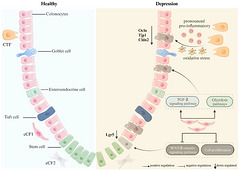

## 1 Introduction

Depressive disorders impose a substantial global burden on public health, representing a leading cause of morbidity and mortality. The World Health Organization projects that by 2030, they will rank first in terms of global disease burden (Malhi et al. 2018). Reflecting this scale, recent prevalence data indicate that approximately 18% of adults report current depression, while 29% report a lifetime history of the condition (Lee et al. 2023). The well‐established concept of bidirectional brain–gut communication involves several interacting pathways, including the autonomic, enteric, and neuroendocrine systems, alongside immune mediators (MacKay et al. 2024). How this central–peripheral crosstalk is reconfigured in disease states, however, warrants further investigation.

Within the colonic mucosa, fibroblasts constitute a core stromal cell (SC) population critical for maintaining epithelial homeostasis (Schuster et al. 2023). Their function is closely linked to their anatomical position along the crypt axis. Crypt‐base fibroblasts localize adjacent to Lgr5‐positive stem cells, whereas crypt‐top fibroblasts reside near the luminal junction (Brügger and Basler 2023). Under physiological conditions, these spatially defined subsets cooperate to establish a longitudinal Wnt and BMP signaling gradient through the secretion of antagonistic molecules. This gradient is essential for the coordinated regulation of stem cell renewal and epithelial differentiation, which underpins intestinal barrier integrity (Gehart and Clevers 2019).

This meticulously balanced microenvironment may be vulnerable to disruption during chronic stress, such as that which characterizes depression. Stress is known to alter the intestinal landscape via neuroendocrine and immune pathways, for instance, through activation of the hypothalamic–pituitary–adrenal (HPA) axis and the release of pro‐inflammatory cytokines (Fazilaty et al. 2021). In response, resident fibroblasts—particularly peri‐cryptal fibroblasts, or crypt‐base fibroblasts, that express PDGFRα and serve as a critical source of Wnt ligands and R‐spondins—can become activated under chronic stress conditions (Brügger and Basler 2023; Degirmenci et al. 2018). This activation leads to phenotypic shifts, including the adoption of a pro‐inflammatory state and alterations in their secretory profile. Such changes involve an increase in inflammatory mediators, such as IL‐6 and CXCL1, and a decrease in reparative signals, defined here as niche‐supporting factors that promote epithelial stem cell maintenance and barrier integrity, including Wnt agonists such as Wnt2, Wnt2b, and Rspo3, as well as BMP antagonists such as Grem1 (Gehart and Clevers 2019; Greicius et al. 2018). These alterations have been shown to contribute to epithelial barrier dysfunction, potentially through downregulation of tight junction proteins, including occludin (Kiecolt‐Glaser et al. 2015). Cumulatively, these changes likely disrupt epithelial homeostasis and may form a key pathological link to the gastrointestinal symptoms and microbiota‐gut–brain axis disturbances observed in depression.

Single‐cell RNA sequencing (scRNA‐seq) has emerged as a powerful tool for resolving cellular heterogeneity within complex tissues and for uncovering molecular insights into pathophysiological processes (Cheng et al. 2024). In intestinal research, it has been instrumental in defining epithelial cell subtypes and in mapping the dynamic states of individual cells under various conditions, thereby advancing our understanding of disease mechanisms and repair responses (Shiau et al. 2024).

In this study, we performed scRNA‐seq on colon tissues obtained from control mice and from mice subjected to chronic unpredictable mild stress (CUMS), a well‐established model of depression. The resulting dataset provides a valuable resource for elucidating the cellular composition, dynamic changes, temporal heterogeneity, and underlying mechanisms involved in colonic injury within the context of depression.

## 2 Materials and Methods

### 2.1 Mice and Treatments

A total of twelve 8‐week‐old male C57BL/6J mice, weighing 20 ± 2 g, were purchased from Beijing HFK Bioscience Co. Ltd. (SCXK (Jing) 2024‐0003) and housed under SPF conditions at the Animal Facility of Hebei Provincial Hospital of Traditional Chinese Medicine (SYXK (Ji) 2023‐012). The housing environment was maintained at 22°C–24°C with 50%–65% relative humidity under a 12 h/12 h light/dark cycle. Mice were randomly allocated to either a control group (CON, *n* = 6) or a model group (M, *n* = 6) using a random number table. Depression‐like behavior was induced in the M group via a 5‐week CUMS protocol, as previously described (Sharma et al. 2024). Briefly, the CUMS paradigm consisted of the following stressors: 24‐h food deprivation, 24‐h water deprivation, 12‐h damp bedding, cage crowding, empty cage, noise and foot shock, 4–6‐h physical restraint, 4‐min tail clipping, 5‐min forced swimming in 4°C water, and 12‐h cage tilt at 45°. Mice in the M group were exposed to two different randomly selected stressors each day, with the same stressor never applied on two consecutive days. After the 5‐week modeling period, behavioral tests were conducted on all animals. Following a 12‐h fast (with free access to water), mice were euthanized via intraperitoneal injection of 150 mg/kg sodium pentobarbital. Colon tissues were then collected for subsequent analysis. All experimental procedures were approved by the IACUC of Hebei Provincial Hospital of Traditional Chinese Medicine (No. IACUC‐HPHCM‐2025009).

### 2.2 Behavioral Assays

Depression‐like phenotypes were evaluated using the sucrose preference test (SPT), open‐field test (OFT), and tail suspension test (TST).

#### 2.2.1 SPT

Mice were first acclimated for 24 h to two bottles of tap water. After 24 h of water deprivation, a 12‐h two‐bottle choice test was conducted, offering one bottle with 1% (w/v) sucrose solution and another with plain water. Bottle positions were switched midway to control for side bias. Sucrose preference was calculated as follows: Sucrose preference (%) = [sucrose intake / (sucrose intake + water intake)] × 100%.

#### 2.2.2 OFT

Locomotor and exploratory activity was evaluated in a black, square open‐field arena (45 × 45 × 30 cm). Each mouse was placed gently in the center of the arena and allowed to explore freely for 5 min. The total distance traveled was recorded and analyzed using Smart 3.0 video tracking software (Rwd Life Science Co. Ltd., Shenzhen, China).

#### 2.2.3 TST

Mice were suspended by the tail with adhesive tape (1–2 cm from the tip) approximately 20–30 cm above the floor. After a 2‐min acclimation period, immobility time was recorded over the subsequent 4 min. Immobility was defined as the absence of any limb or body movement.

### 2.3 Single‐Cell Suspension Preparation

Colon segments were dissected, minced, and processed into a single‐cell suspension using an established protocol (Reichard and Asosingh 2019). Briefly, Colon tissues were immediately placed in ice‐cold PBS after dissection, opened longitudinally, and gently scraped to remove feces and mucus. The tissues were then rinsed 3–5 times with ice‐cold PBS until the wash solution became clear. The cleaned tissues were minced into 1–2 mm^3^ pieces and transferred to digestion buffer containing Collagenase IV (2 mg/mL) and DNase I (0.1 mg/mL) in HBSS. Digestion was first carried out in a 37°C water bath with gentle shaking for 10 min, followed by further digestion in a thermostatic shaker at 37°C and 120 rpm for 20 min. The digestion was immediately stopped on ice, and the tissue suspension was gently pipetted 5–10 times using a serological pipette to facilitate cell dissociation. The suspension was then filtered through a 70 µm cell strainer. The filtrate was centrifuged at 400 × *g* for 5 min at 4°C. The pellet was optionally treated with red blood cell lysis buffer to remove erythrocytes, and then resuspended in PBS containing 0.04% bovine serum albumin (BSA). Cell viability was assessed by trypan blue staining, and only samples with viability > 70% were used for subsequent library preparation. The final cell concentration was adjusted to 500–2000 cells/µL in a minimum volume of 200 µL. The entire procedure from tissue harvest to cell loading was completed within 2 h.

### 2.4 ScRNA‐Seq Protocol

Colon tissues were collected from CON and M groups (*n* = 3/group). A total of 21,967 cells (11,199 from the CON group and 10,768 from the M group) were used for scRNA‐seq library construction with the SeekOne DD Single Cell 3′ Transcriptome Kit (SEEKGENE, Beijing, China), following the manufacturer's protocol. The resulting barcoded scRNA‐seq libraries were prepared for high‐throughput sequencing and subjected to quality control before sequencing on an Illumina NovaSeq 6000 platform (Illumina, San Diego, CA, USA). Sequencing was performed to a depth of at least 50,000 reads per cell to ensure data reliability.

Raw sequencing reads were processed with fastp to remove low‐quality reads. The filtered data were then analyzed using SeekSoul Tools to generate a gene expression matrix. Subsequent analyses, including cell clustering, differential gene expression, and enrichment analysis, were performed on the SeekSoul Online cloud platform (https://seeksoul.online/index.html).

Prior to analysis, low‐quality cells were excluded based on the following criteria: cells with fewer than 200 or more than 10,000 detected genes, or with mitochondrial gene content exceeding 40% (Sun et al. 2025). After quality filtering, 16,641 high‐quality cells (8080 from CON group and 8561 from M group) were retained for downstream analysis. Unsupervised clustering was used throughout the analysis to enable unbiased identification of cell populations without predefined markers. This data‐driven approach is standard for exploratory scRNA‐seq studies, as it allows discovery of potentially novel or disease‐relevant cell subsets that might be missed by supervised methods (Kiselev et al. 2019; Luecken and Theis 2019).

### 2.5 Pseudotime Analysis

Cell differentiation trajectories were constructed using Monocle2. The analysis input was the UMI count matrix, with trajectory inference based on significant marker genes identified by Seurat (*q* < 10^−4^) (Z. Tian and Yang 2023).

### 2.6 Differentially Expressed Gene Analysis

Differentially expressed genes (DEGs) between cell clusters were identified using the FindMarkers function in Seurat. A Wilcoxon rank‐sum test with Bonferroni correction was applied. The false discovery rate (FDR) was controlled using the Benjamini–Hochberg procedure, with significance defined as *q* ≤ 0.01, absolute log2 fold change ≥ 0.25, and gene detection in > 10% of cells per cluster (Shuken and McNerney 2023).

### 2.7 Gene Ontology and Pathway Assays

Cell subtypes were annotated using established marker genes. Gene Ontology (GO) and Kyoto Encyclopedia of Genes and Genomes (KEGG) pathway enrichment analyses were performed. Significant terms (*q* < 0.05) were identified using Fisher's exact test with FDR adjustment (Yu et al. 2012).

### 2.8 Gene Set Variation Analysis

Gene Set Variation Analysis (GSVA) was performed on the SeekSoul Online platform using default parameters. The analysis used the MSigDB hallmark gene set collection (H). Gene set enrichment scores were calculated per cell based on normalized expression data, and differences between CON and M groups were assessed using Wilcoxon rank‐sum test. Results are presented as *Z*‐score‐normalized heatmaps.

### 2.9 Cell‒Cell Communication Analysis

Intercellular communication analysis is a pivotal component in the interpretation of scRNA‐seq data. To explore ligand‐receptor‐mediated interactions among distinct cell populations, we utilized CellPhoneDB version 2.0, with particular emphasis on the most statistically significant ligand‐receptor pairs (Top 50 pairs; *p* < 0.05) (Vento‐Tormo et al. 2018).

### 2.10 Intestinal Organoid Isolation, Culture, and Treatment

Intestinal crypts were isolated from the colons of C57BL/6J mice in both CON and M groups using established protocols with minor modifications (J. Liang, Dai, et al. 2024). Briefly, intestinal tissues were dissected, rinsed in ice‐cold PBS, sectioned into 5‐mm fragments, and washed repeatedly until supernatants appeared clear. The tissue fragments were then incubated in digestion buffer at 4°C for 15 min under continuous shaking. After removal of the digestion solution, the tissues were resuspended in PBS containing 1% BSA and mechanically dissociated by passage through a 70‐µm cell strainer (Human BKMAM, Shenzhen, China) to remove residual villous debris. Intact crypts were pelleted by centrifugation at 200 × *g*, resuspended in Matrigel (Corning, Glendale, AZ, USA, 00625001), and seeded into 24‐well plates. Following polymerization at 37°C for 15 min, each well was overlaid with 500 µL of IntestiCult Organoid Growth Medium (STEMCELL Technologies, Vancouver, BC, Canada, 06003). Cultures were maintained at 37°C in a humidified incubator with 5% CO_2_. Organoid development was monitored daily via brightfield microscopy using a Leica DMi1 microscope (Wetzlar, Germany) over a period of 10 days.

### 2.11 Immunofluorescence (IF) Analysis

The IF analysis was performed as previously described (Luo et al. 2024). Specifically, organoids cultured in Matrigel droplets were washed with DPBS and fixed with 4% paraformaldehyde (PFA) for 30 min at room temperature until the Matrigel completely disintegrated. After three washes with DPBS, the samples were permeabilized with DPBS containing 0.1% Tween‐20 and 0.2% Triton X‐100 for 15 min. Nonspecific binding was blocked with 5% BSA in DPBS for 1 h at room temperature. The samples were then incubated with primary antibodies diluted in DPBS containing 1% BSA overnight at 4°C (approximately 16–18 h). The primary antibodies and dilutions used were: rabbit anti‐Ki67 (Abcam, ab15580, 1:200), rabbit anti‐Lgr5 (Abgent, AP2020b, 1:100), and rabbit anti‐ZO‐1 (Thermo Fisher Scientific, 61‐7300, 1:100). After three washes with DPBS (5 min each), the samples were incubated with HRP‐conjugated goat anti‐rabbit secondary antibody (Abcam, ab205718, 1:500) for 1 h at room temperature. Following additional washes, signal amplification was performed using a CY3‐conjugated tyramide signal amplification (TSA) kit (PerkinElmer, NEL744001KT) according to the manufacturer's instructions. Nuclei were counterstained with DAPI (1 µg/mL) for 5 min. Finally, the samples were mounted with an antifade mounting medium, and images were acquired using a Nikon Eclipse C1 laser scanning confocal microscope (Nikon Instruments Inc., Tokyo, Japan).

### 2.12 Statistical Analysis

All data processing, statistical analysis, and visualization were performed using GraphPad Prism (version 8.0.2). Normality was assessed using the Shapiro–Wilk test. For comparisons between two groups, if data met normality and equal variance assumptions, an unpaired two‐tailed Student's *t*‐test was used; otherwise, the Mann–Whitney *U* test was applied. All statistical tests were two‐sided, and statistical significance was defined as *p* < 0.05.

## 3 Results

### 3.1 Depression‐Like Behaviors Induced by CUMS

**FIGURE 1:**
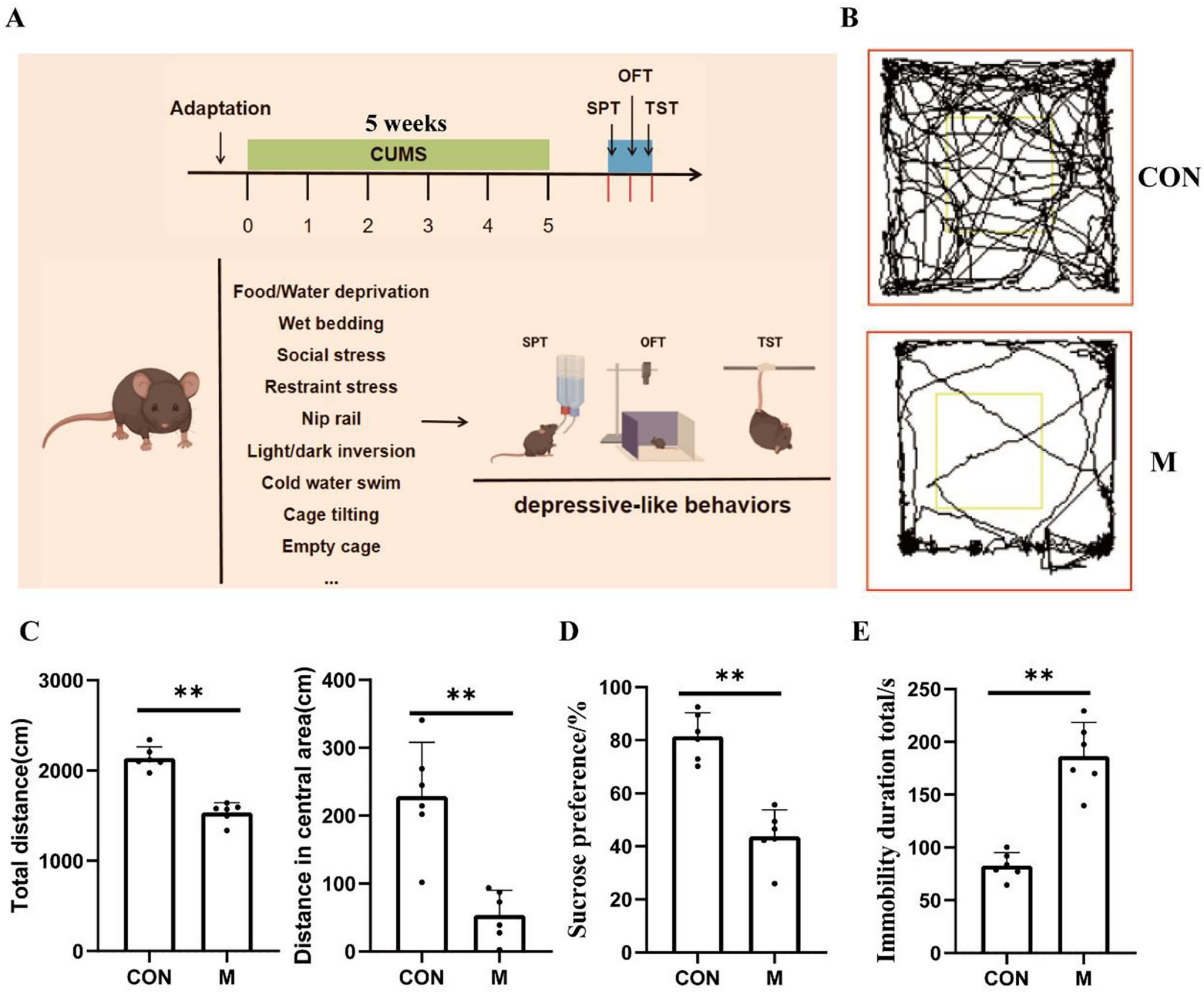
CUMS induces depressive‐like behaviors in mice. (A) Experimental timeline of the CUMS paradigm and behavioral assessments. (B) Representative movement traces of mice in the OFT. (C) Quantification of total distance traveled and distance in the central area during OFT, (D) sucrose preference in the SPT, and (E) immobility time in the TST. Data are presented as mean ± SEM with *n* = 6 mice per group. Student's *t*‐test was used for comparisons between the two groups. **p < 0.01 vs. CON group. CUMS: chronic unpredictable mild stress; OFT: open‐field test; SPT: sucrose preference test; TST: tail suspension test.

The experimental timeline for the CUMS model and subsequent behavioral assessments is shown in Figure 1A. Mice exposed to the 5‐week CUMS paradigm displayed robust behavioral deficits indicative of depression.

#### 3.1.1 OFT

Compared with CON group, the mice in the M group showed significantly reduced locomotor activity. The total distance traveled was 2138.9 ± 123.6 cm in the CON group and 1536.4 ± 108.4 cm in the M group (t (10) = 8.98, *p* < 0.01). The distance traveled in the central arena was 228.8 ± 79.3 cm in the CON group and 53.7 ± 36.3 cm in the M group (t(10) = 4.92, *p* < 0.01) (Figure 1B,C).

#### 3.1.2 SPT

Anhedonia, a core symptom of major depressive disorder (Duman 2007), was assessed by SPT. The sucrose preference percentage was 81.5 ± 8.8% in the CON group and 43.8 ± 10.0% in the M group, indicating a marked reduction in the M group (t(10) = 6.92, *p* < 0.01) (Figure 1D).

#### 3.1.3 TST

The immobility time in the TST was significantly increased in the M group. The CON group showed an immobility time of 82.8 ± 12.4 s, whereas the M group showed 186.5 ± 32.0 s (t(10) = 7.41, *p* < 0.01) (Figure 1E).

These results demonstrate that the 5‐week CUMS protocol successfully induced depressive‐like behaviors in mice.

### 3.2 Overview of Colon Cells

**FIGURE 2:**
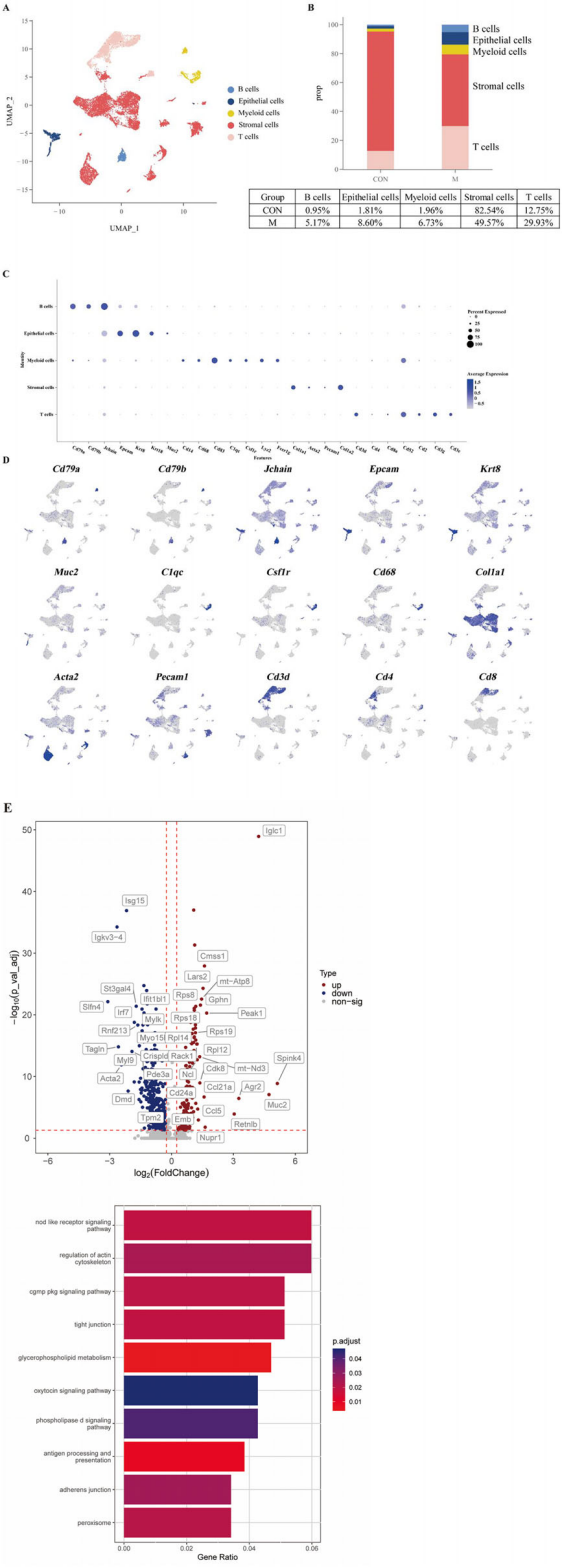
Single‐cell profiling of colonic cellular composition in a mouse model of depression. (A) UMAP plot annotated with the five major cellular compartments identified in this study with *n* = 3 mice per group. (B) Stacked bar charts showing the relative proportions of major cell types in the Control and M groups. (C) Dot plot showing the annotation of cell subtypes based on established marker genes. (D) Expression distribution of key signature genes across the identified cell types (color intensity indicates average expression level). (E) Differentially expressed genes in the colons of depression model mice and their enriched KEGG pathways.

To define the cellular alterations in the colon under depressive conditions, we performed scRNA‐seq on colon tissues from CON group and M group. After quality control, 16,641 high‐quality cells were analyzed. Unsupervised clustering was performed based on transcriptional profiles. Using established marker genes (Tao et al. 2025; Yin et al. 2023), we annotated the clusters and identified five major cell lineages. (Figure 2A). To account for differences in total cell recovery between the CON and M groups, comparative data are presented as the relative proportion of each cell type (Figure 2B).

This proportional analysis revealed a marked shift in cellular composition. The proportion of SCs was substantially lower in the M group, at 49.57%, compared with 82.54% in CON group. Conversely, the relative proportions of T cells, B cells, epithelial cells, and myeloid cells were all elevated in the depression model. The identity of these five major clusters was confirmed by canonical marker gene expression. B cells were defined by expression of *Cd79a*, *Cd79b*, and *Jchain*; epithelial cells by *Epcam*, *Krt8*, and *Muc2*; myeloid cells by *C1qc*, *Csf1r*, and *Cd68*; SCs by specific expression of *Col1a1*, *Acta2*, and *Pecam1*; and T cells by *Cd3d*, *Cd4*, and *Cd8* (Figure 2C,D).

To visualize the global transcriptional differences between CON and M groups, we generated a volcano plot (Figure 2E). Within the epithelial compartment of the depression model, we observed distinct transcriptional changes. Interferon‐responsive genes, including *Isg15* (Mishra et al. 2021) and *Slfn4* (Zhang et al. 2025), were downregulated, as was *Tagln*, a gene implicated in regulating smooth muscle motility (Chakraborty et al. 2019). In contrast, the expression of key genes essential for mucosal barrier defense was significantly upregulated. These included *Spink4* (Y. Wang et al. 2024), *a marker of secretory progenitor cells that give rise to Muc2^+^ lineages*, and *Muc2* (Gorman et al. 2025), the major gel‐forming mucin constituting the primary mucus scaffold.

KEGG pathway enrichment analysis of all DEGs further clarified the involved biological processes. These genes were significantly enriched in core pathways related to innate immune sensing, such as the NOD‐like receptor signaling pathway; cellular structural remodeling, including regulation of the actin cytoskeleton and the tight junction pathway; cell signal transduction, such as the cGMP‐PKG and oxytocin signaling pathways; and metabolic processes, like glycerophospholipid metabolism (Figure 2E, Figure S2).

### 3.3 Functional Differentiation of Colonic Epithelial Cells Is Disrupted in Depression

**FIGURE 3:**
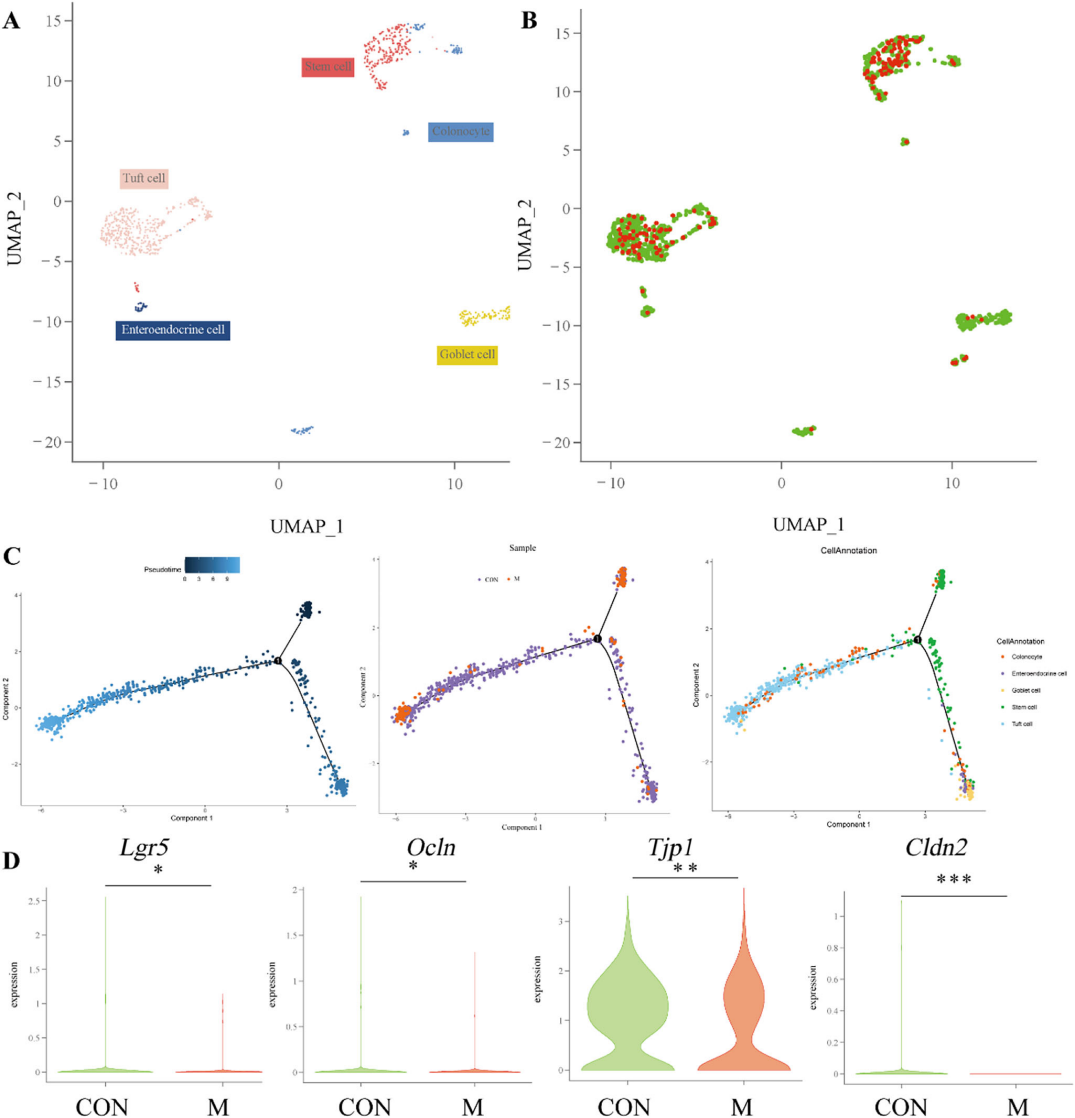
Heterogeneity of colonic epithelial cells in a mouse model of depression. (A) UMAP visualization of epithelial cells colored by cluster identity. (B) Proportion of epithelial cell subsets in the CON and M groups (green: CON group; red: M group). (C) Predicted differentiation trajectory of colonic epithelial cells. (D) Expression levels of stem cell markers in the CON and M groups. Statistical comparisons were performed using Wilcoxon rank‐sum test; *p < 0.05, **p < 0.01, and ***p < 0.001 vs. CON group.

Unsupervised clustering resolved colonic epithelial cells into five principal subsets: stem cells, colonocytes, tuft cells, enteroendocrine cells, and goblet cells (Figure 3A). The absolute number of cells within each epithelial subset was consistently reduced in the colon of M group compared with CON group (Figure 3B).

To elucidate the mechanism underlying this cellular depletion, we performed pseudotime trajectory analysis. In CON group, epithelial cells followed a continuous and uniform trajectory from a stem cell state toward distinct terminally differentiated fates. This orderly progression was severely disrupted in M group. Cells aberrantly accumulated at the two extremes of the trajectory, clustering around an undifferentiated, stem‐like state at one end and a specialized tuft cell state at the other, instead of forming a continuum (Figure 3C). This dichotomous accumulation indicates concurrent impairments in both the initiation and completion of the epithelial differentiation program.

Corroborating this dysregulated state, epithelial cells from depressed mice exhibited significantly lower expression of key functional genes. This included the downregulation of the canonical stem cell marker *Lgr5* (*p* < 0.05), alongside the core tight junction genes *Ocln* (*p* < 0.01), *Tjp1*(encoding ZO‐1, *p* < 0.01), and *Cldn2*(*p* < 0.001), relative to CON group (Figure 3D).

### 3.4 Stromal Cells Adopt a Pro‐Inflammatory/Dysfunctional Phenotype in Depression

**FIGURE 4:**
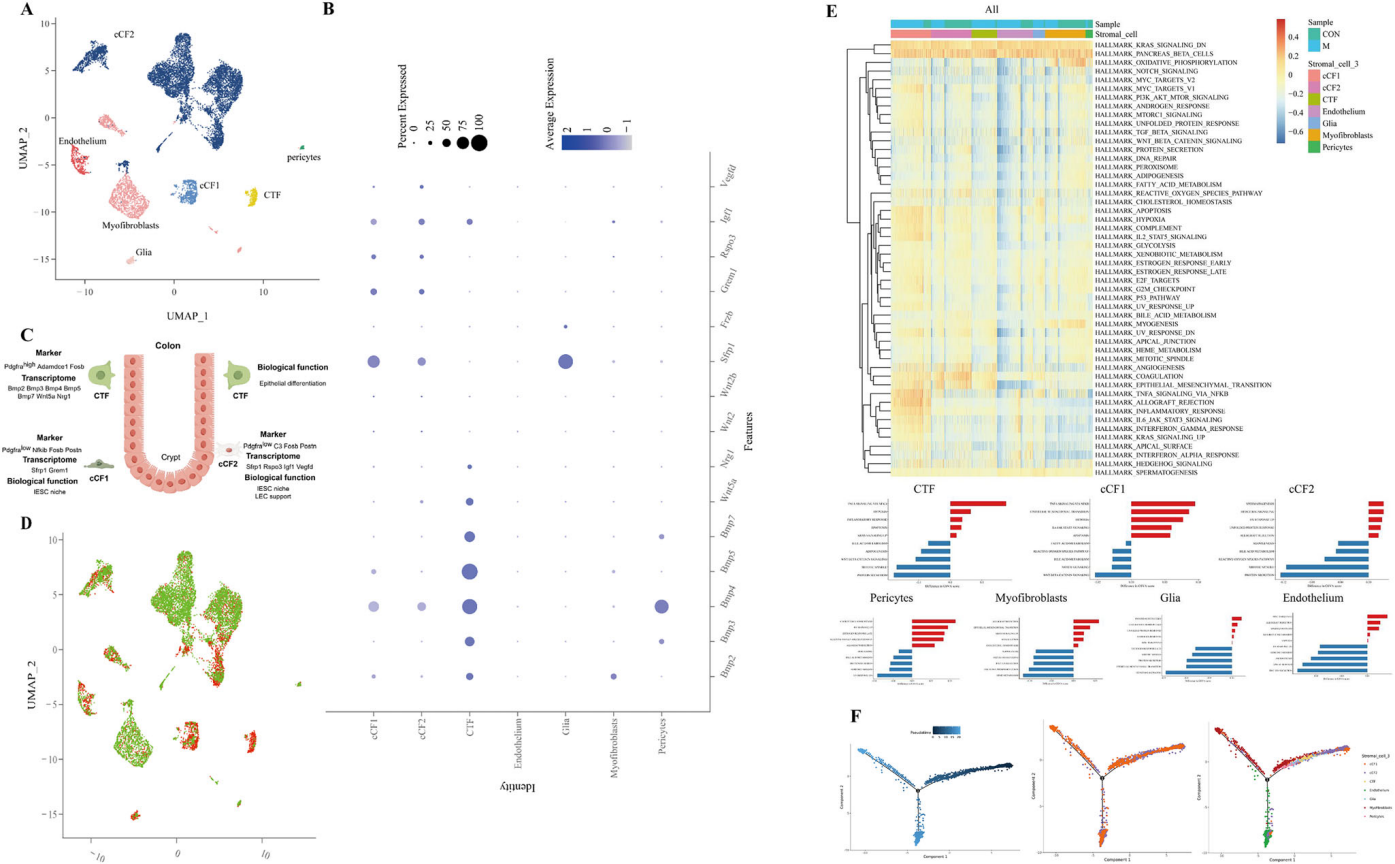
Heterogeneity of colonic stromal cells in a mouse model of depression. (A) UMAP visualization of stromal cells colored by cluster identity. (B) Annotation of seven transcriptionally distinct stromal subtypes based on known marker genes. (C) Schematic diagram illustrating the spatial organization and classification of major colonic fibroblast subsets. (D) Proportional abundance of stromal cell subsets in CON and M groups. Green represents the CON group and red represents the M group. (E) Heatmap depicting GSVA‐derived pathway activity scores (*Z*‐scores) across stromal cell subsets. Red indicates pathway enrichment in the M group relative to CON, and blue indicates suppression. (F) Pseudotime trajectory analysis reconstructing the differentiation dynamics of colonic stromal cells.

Colonic SCs were further classified into seven transcriptionally distinct subpopulations (Figure 4A). Among these, the cCF1, cCF2(colonic crypt‐bottom fibroblast 2), and CTF subsets displayed unique secretory profiles linked to morphogen signaling. Specifically, cCF1 and cCF2 subsets were characterized by high expression of Wnt signaling antagonists such as *Sfrp1* and *Grem1*, along with *Bmp4*. In contrast, CTF cells specifically expressed elevated levels of multiple BMP ligands, including *Bmp2*, *Bmp3*, *Bmp4*, *Bmp5*, and *Bmp7*, as well as the noncanonical Wnt ligand *Wnt5a* (Figure 4B). These three subsets constitute the major colonic fibroblast populations, with cCF1 and cCF2 subsets localized to the crypt base and CTF subset residing at the crypt apex, as supported by prior work (Brügger and Basler 2023) (Figure 4C). To further characterize the functional identity of each stromal subcluster, we performed systematic annotation based on their specifically expressed marker genes. cCF1 and cCF2 are both localized to the crypt base and highly express Wnt antagonists including Sfrp1 and Grem1, along with the BMP ligand Bmp4. In addition, cCF1 specifically expresses elevated levels of Tgfb1 and Col1a1, whereas cCF2 is marked by high expression of Cd34 and Pdgfra. These features suggest that both cCF1 and cCF2 contribute to the stem cell niche at the crypt base, with cCF1 possessing a stronger pro‐fibrotic potential. CTF resides at the crypt apex and specifically expresses multiple BMP ligands, including Bmp2, Bmp3, Bmp4, Bmp5, and Bmp7, together with the non‐canonical Wnt ligand Wnt5a. This expression profile supports its role in promoting epithelial terminal differentiation through BMP‐mediated signaling at the luminal compartment. The remaining stromal subclusters each display distinct marker signatures and are assigned to non‐fibroblast lineages. Endothelial cells are defined by expression of Pecam1, Cd34, and Emcn, contributing to vascular homeostasis. Glial cells express Plp1, Sox10, and Mbp, representing the enteric glial component. Myofibroblasts show high expression of Acta2, Tagln, and Col3a1, consistent with a smooth muscle‐like contractile function. Pericytes are characterized by expression of Kcnj8, Abcc9, and Rgs5, participating in microvascular stabilization. Collectively, cCF1 and cCF2 function as crypt‐base niche‐supporting cells, while CTF serves as a crypt‐top differentiation‐regulating population. Endothelial cells, glial cells, myofibroblasts, and pericytes are primarily responsible for vascular, neural, and stromal structural maintenance, respectively.

In the M group, the proportional abundances of the CTF and cCF1 subsets were significantly increased compared with CON group (Figure 4D), implicating them as potential disease‐specific effector populations.

To define the functional states of these expanded subsets, we performed GSVA separately for each of the seven stromal subclusters. This analysis revealed that the transcriptional reprogramming induced by depression was highly subset‐specific rather than globally uniform across all stromal populations (Figure 4E). The cCF1 subset exhibited significantly increased enrichment scores for TNF‐α/NF‐κB signaling, epithelial‐mesenchymal transition (EMT), hypoxia, and IL‐6/JAK/STAT3 signaling pathways in the M group compared with the CON group, accompanied by a further suppression of Wnt/β‐catenin signaling. This pattern suggests a phenotypic shift toward a pro‐fibrotic, anti‐proliferative state. The CTF subset displayed a pronounced pro‐inflammatory and oxidative stress signature, marked by elevated enrichment of TNF‐α/NF‐κB signaling, hypoxia, and inflammatory response pathways, together with marked downregulation of Wnt/β‐catenin signaling, mitotic spindle, and protein secretion pathways. Given its anatomical location at the crypt apex, this functional shift is consistent with a potential role in modulating epithelial differentiation and barrier function. The cCF2 subset, although exhibiting relatively more moderate changes, also showed reduced enrichment of mitotic spindle and protein secretion pathways, suggesting impaired proliferative activity. The endothelial cell subset predominantly exhibited downregulation of angiogenesis, apical surface, and protein secretion pathways, implying that depression may affect colonic vascular homeostasis. The glia and myofibroblast subsets displayed more heterogeneous patterns. Myofibroblasts showed concurrent upregulation of EMT and downregulation of oxidative phosphorylation pathways, while glial cells mainly exhibited suppression of Hedgehog signaling and EMT pathways. The pericyte subset showed the most distinct response pattern, with increased enrichment of cholesterol homeostasis, UV response, and reactive oxygen species pathways in the M group.

Pseudotime trajectory analysis revealed the dynamic relationships among these subsets (Figure 4F). Under depressive conditions, the stromal differentiation landscape was skewed, with an increased accumulation of cells toward CTF, cCF1, endothelial, and glial cell states, and a concomitant reduction in the cCF2 state. This shift indicates that depression systematically reshapes SC fate decisions, driving a collective transition toward specific dysfunctional phenotypes rather than merely expanding pre‐existing populations. This altered trajectory aligns with the changes in cellular proportions observed in the depressed state (Figure S3).

### 3.5 Depression Disrupts the Core Stromal–Epithelial Cell Communication Network

**FIGURE 5:**
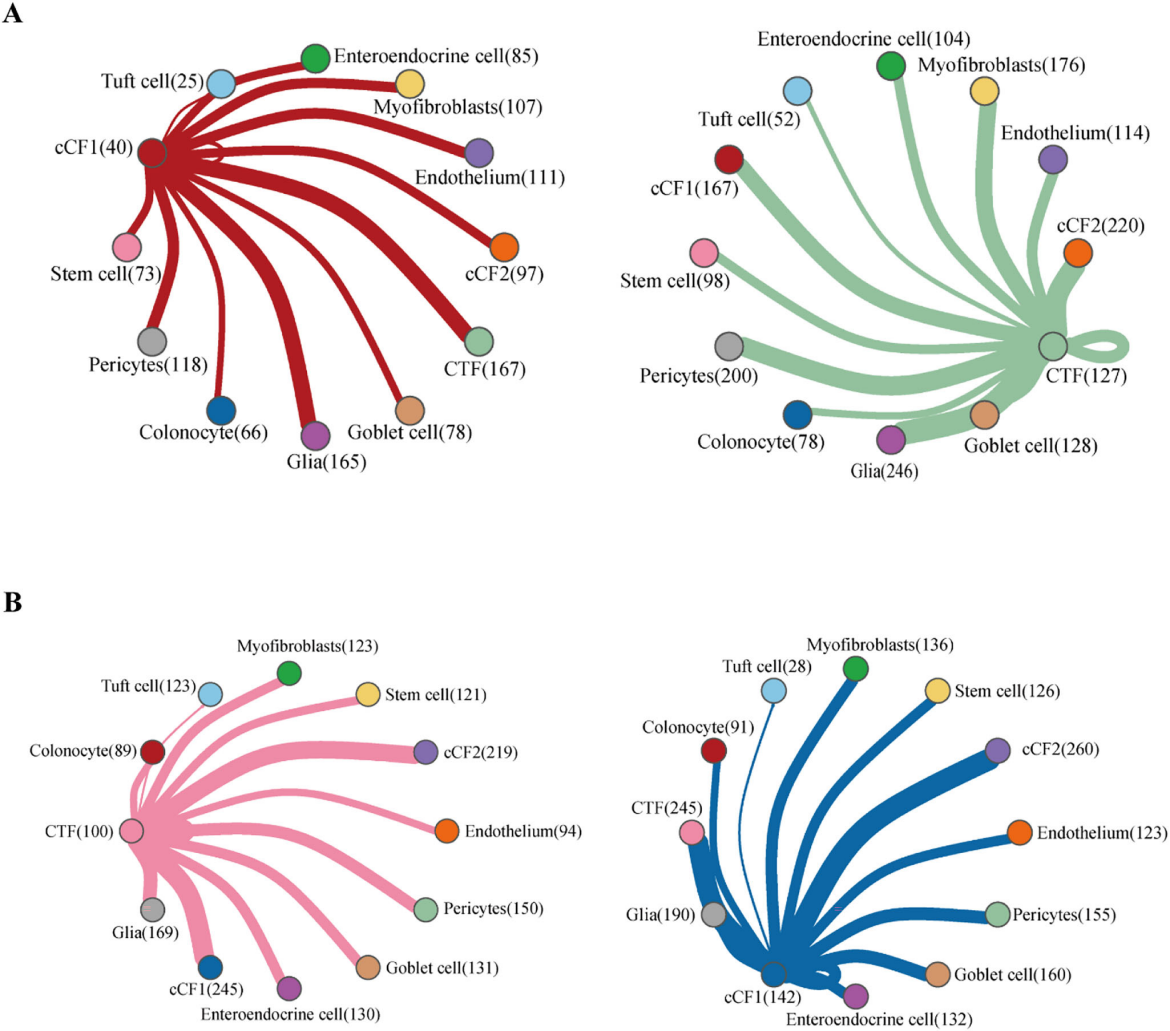
Intercellular communication analysis between colonic cell subsets in CON and M groups. Network diagrams showing ligand–receptor interactions between cell types, with nodes representing cell subsets and edge thickness representing the number of statistically significant ligand–receptor pairs between each pair of cell types. Only pairs with *p* < 0.05 were considered significant and are displayed. (A) CON group; (B) M group. Compared with the CON group, the M group exhibits enhanced interactions between epithelial stem cells and the expanded stromal subsets cCF1 and CTF, as indicated by increased edge thickness. The analysis was performed using CellPhoneDB version 2.0.

To evaluate changes in cellular communication under depressive conditions, we performed a systematic analysis of cell–cell interactions. The resulting interaction matrices and network diagrams revealed a profound rewiring of the colonic signaling landscape in depression. In the CON group (Figure 5A), cell–cell interactions were relatively sparse and evenly distributed across cell types. The cCF1 and CTF exhibited limited outgoing signaling toward epithelial stem cells. In striking contrast, the M group (Figure 5B) displayed a globally intensified interaction network. The expanded stromal subsets cCF1 and CTF established dramatically enhanced signaling connections with epithelial stem cells. Specifically, cCF1 showed a substantial increase in outgoing interactions toward epithelial stem cells, while CTF similarly strengthened its outgoing signaling to epithelial stem cells.

### 3.6 Depression Reduces the Abundance of Ki67, Lgr5, and ZO‐1 Cells in the Intestine

**FIGURE 6:**
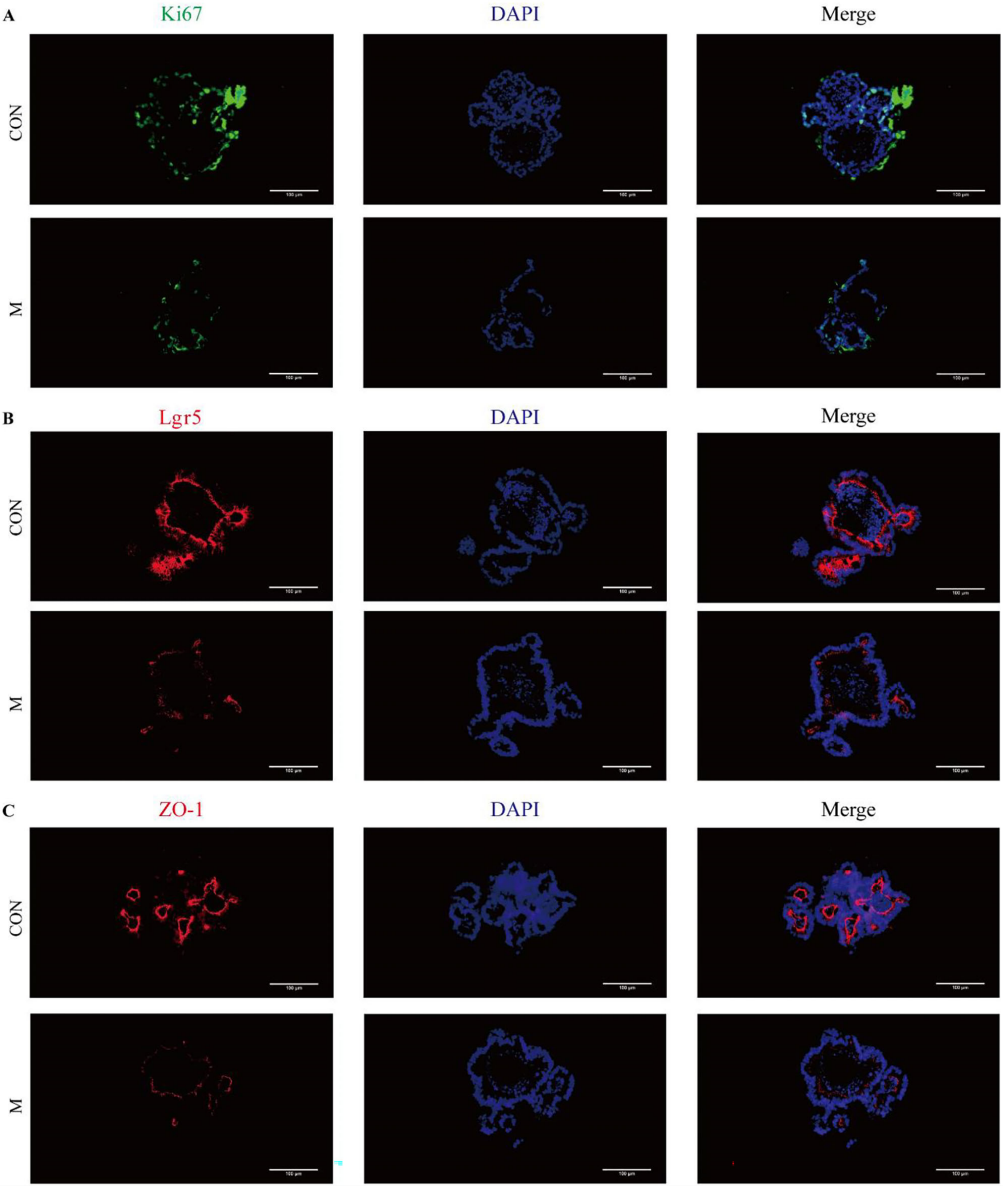
Immunofluorescence staining of intestinal organoids. (A) Staining for Ki67 (green). (B) Staining for Lgr5 (red). (C) Staining for ZO‐1 (red). Nuclei were counterstained with DAPI (blue). Scale bar: 100 µm.

To functionally assess the impact of depression on intestinal homeostasis, we established and analyzed intestinal organoids. IF staining of organoid sections revealed a reduced in Ki67‐positive cells in organoids derived from the M group compared with the CON group. Staining for Lgr5 and ZO‐1 also appeared less intense in the M group relative to the CON group (Figure 6A–C).

## 4 Discussion

Depression represents a major cause of disability worldwide (Roest et al. 2021). Increasing attention has been focused on the brain–gut axis and its role in disease progression. This complex interplay involves a multifaceted regulatory network encompassing neuroendocrine pathways—including the HPA axis—the central nervous system, the peripheral nervous system, and the autonomic nervous system. In mouse models of colitis in remission, activation of these pathways can suppress vagal responses, subsequently inducing depression‐like behaviors and reigniting intestinal inflammation (Ghia et al. 2009). This interaction appears to be bidirectional, suggesting that the clinical symptoms of common psychiatric disorders are linked to the gastrointestinal tract at a pathophysiological level, potentially creating a mutually reinforcing cycle (Martin‐Subero et al. 2016). Intestinal SCs not only provide structural support to the gut wall but also, as recent studies indicate, play crucial roles in immune regulation, tissue repair, and regeneration (Chen et al. 2024). However, the contribution of SCs to intestinal alterations in depression remains unclear.

This study combines a CUMS mouse model of depression with single‐cell transcriptomics of colonic tissue, bioinformatic analysis, and intestinal organoid validation. It suggests a profound, multidimensional remodeling of the colon in depression, encompassing cellular composition, differentiation programs, intercellular communication, and tissue regenerative capacity. Key findings include: the disruption of the epithelial differentiation trajectory alongside downregulation of stem cell and barrier genes; the pathological expansion and phenotypic shift of specific stromal subsets, notably pro‐fibrotic cCF1 and pro‐inflammatory CTF cells; a significantly enhanced communication network between these disease‐specific SCs and epithelial stem cells; and the in vivo depletion of barrier‐relevant cells paired with impaired ex vivo organoid growth. Of note, the higher relative proportion of epithelial cells in the M group despite their lower absolute number reflects a passive “dilution effect” following the marked reduction in SC abundance, rather than true epithelial expansion. Together, these data provide a single‐cell atlas of the colon in depression, suggesting a link between the disease and specific, dysfunctional stromal subpopulations and aberrant niche signaling, thereby establishing a novel cellular framework for understanding peripheral gut–brain axis mechanisms.

Our data point to a potentially critical role for the functional heterogeneity of colonic SCs in depression. While stress‐related gut symptoms are often attributed to neuroendocrine (Zeng et al. 2024; C. Liang, Wei, et al. 2024) or immune activation (Huang et al. 2025; Xia et al. 2025; Liu et al. 2024), or to microbial dysbiosis (Valles‐Colomer et al. 2019; Jach et al. 2023), our data suggest that SCs may play a more active and complex role as architectural and signaling hubs of the tissue microenvironment. The cCF1 subset appeared to exhibit an anti‐proliferative, pro‐fibrotic phenotype, characterized by enhanced TGF‐β and suppressed Wnt/β‐catenin signaling, which may relate to reported intestinal dysmotility (P. Tian et al. 2023) and fibrotic tendencies in depression (Y.‐Q. Tian et al. 2022). In contrast, the CTF subset acquired a strong pro‐inflammatory and oxidative stress signature. Given its anatomical position at the crypt apex (Brügger and Basler 2023), CTF cells are strategically located to directly influence epithelial differentiation and barrier function. The expansion of these subsets and the reshaping of the stromal differentiation trajectory suggest that depression induces a profound stromal “reprogramming,” moving beyond the paradigm of passive immune cell infiltration.

The dysregulation of epithelial–stromal crosstalk may represent a central mechanistic link between stress and intestinal dysfunction. We found that enhanced interaction signals originated predominantly from the expanded cCF1 and CTF subsets and targeted epithelial stem cells. This suggests a possible mechanistic basis for the observed epithelial deficits—aberrant stromal signals, such as Wnt‐inhibitory cues from cCF1 and pro‐inflammatory/noncanonical Wnt signals from CTF, likely collectively disrupt stem cell homeostasis and normal differentiation (Brügger and Basler 2023). This model of precise cell–cell interaction imbalance advances beyond prior views that vaguely attributed gut dysfunction to generalized inflammation or stress hormones (Kiecolt‐Glaser et al. 2015; T. Wang et al. 2023), pointing to specific intercellular communication pathways as potential therapeutic targets.

Our findings extend and contextualize existing literature. Clinical and preclinical studies have documented gut barrier weakening, inflammation, and dysbiosis in depression (Medina‐Rodríguez et al. 2024; Trzeciak and Herbet 2021; Pellegrini et al. 2023). Single‐cell analysis provides direct cellular evidence and points to the specific stromal subsets that may drive these changes. For instance, the downregulation of tight junction proteins we observed aligns with reports of increased intestinal permeability in patients (Morena et al. 2025; Stevens et al. 2018). Of note, Spink4, which labels secretory progenitor cells committed to the Muc2^+^ goblet cell lineage, was upregulated together with Muc2 in the colons of depressed mice. This coordinated upregulation might represent an adaptive attempt to strengthen the mucus barrier under chronic stress, although the functional consequences of this change require further investigation. The pro‐inflammatory shift in SCs resonates with the systemic low‐grade inflammation observed in depression, suggesting a process that is amplified and specialized locally within the gut. Importantly, the prominence of SCs contrasts with the immune‐centric pathology of classical inflammatory bowel disease, suggesting the possibility of a unique stroma‐centric pathology in depression‐related gut alterations.

This study has several limitations that outline future directions. First, while we resolved cellular composition at single‐cell resolution, the dissociation process sacrificed spatial context. Although supported by in silico predictions and organoid data, the precise in situ localization and proximity of subsets like crypt‐top fibroblasts to epithelial stem cells require direct validation using multiplex imaging or spatial transcriptomics. Second, while we reveal strong correlations, further work is needed to establish causality. The upstream drivers of stromal reprogramming‐be they systemic cytokines, neural inputs, or microbial metabolites‐remain unidentified. Definitive proof will require demonstrating that specific ablation or activation of cCF1 or CTF subsets in vivo is sufficient to induce or rescue the epithelial phenotype, utilizing tools like lineage tracing and conditional knockout models. Third, our study primarily examines the downstream effects of brain stress on the gut. The critical reciprocal question‐how this remodeled intestinal microenvironment feeds back to the central nervous system to potentially sustain or exacerbate depressive states‐remains open. Addressing this bidirectional loop will require more complex experimental designs.

It is noteworthy that previous studies have implicated alterations in the intestinal transit‑amplifying (TA) cell compartment under chronic stress conditions, which may impact epithelial renewal (Chaves‐Pérez et al. 2022; Gehart and Clevers 2019). In our scRNA‑seq dataset, we were unable to definitively resolve TA cell subsets due to the continuum of proliferative states and the lack of exclusive markers. Nonetheless, the possibility of TA cell dysregulation in the M group cannot be excluded. Future investigations using lineage tracing or flow cytometry‑based analysis of the proliferative zone will be required to address this question.

**FIGURE 7:**
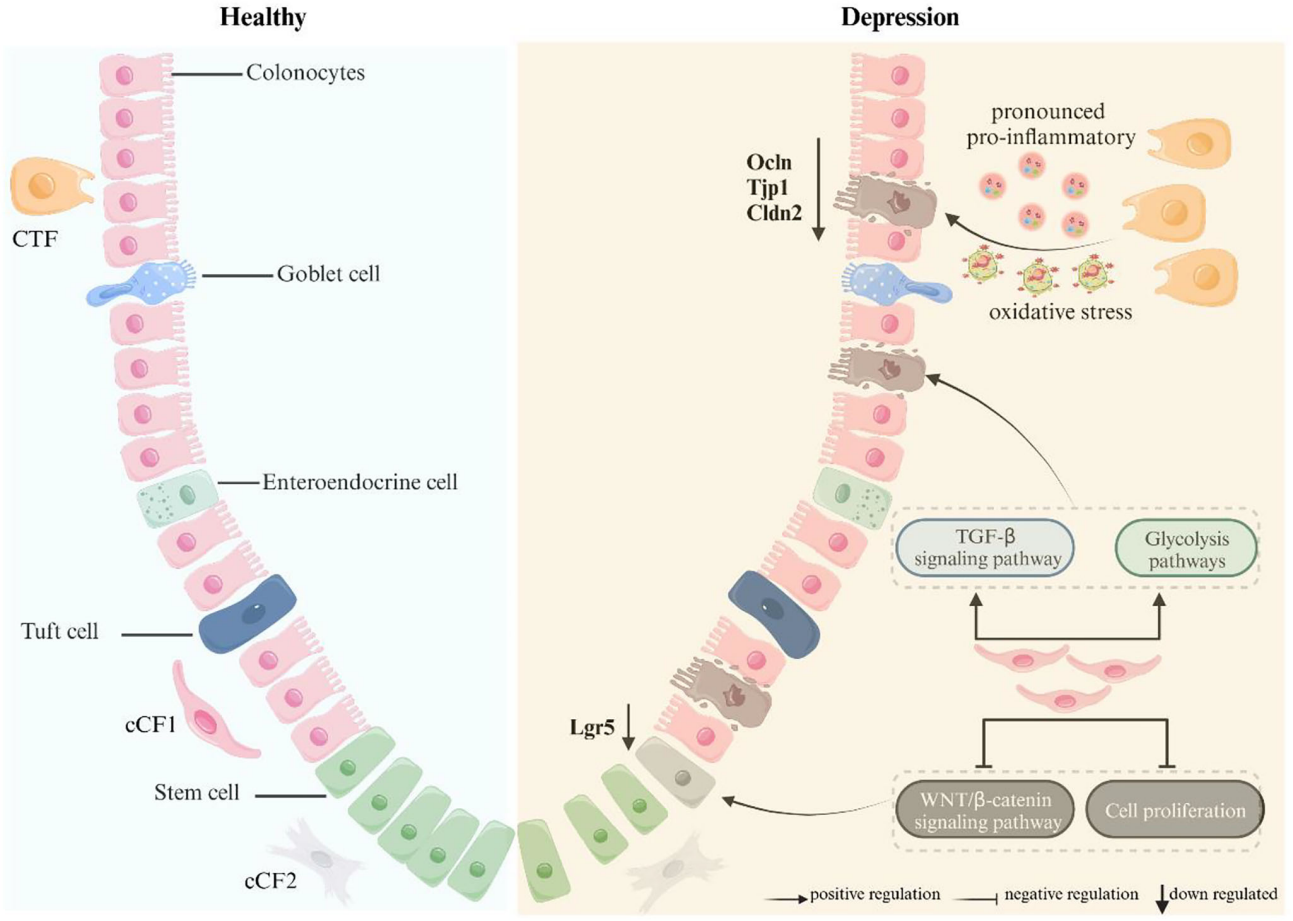
Schematic representation of how disrupted stromal–epithelial crosstalk impairs gut barrier in depression. Depression remodels the colonic stromal niche, driving expansion of subsets characterized by pro‐fibrotic cCF1 and pro‐inflammatory CTF cells. Through dysregulated paracrine signaling involving enhanced TGF‐β and suppressed Wnt pathways, these cells disrupt epithelial homeostasis, which impairs stem cell function, breaks differentiation trajectories, and compromises barrier integrity via downregulation of key tight junction proteins.

In conclusion, this work proposes a novel pathophysiological landscape in the colon, centered on the functional reprogramming of SCs and triggered by chronic stress (Figure 7). It suggests that depression may not solely be a central nervous system disorder but also an instigator of organized cellular shifts in peripheral organs. Targeting these disease‐specific stromal subsets or their signaling pathways may offer new therapeutic avenues for alleviating depression‐associated gastrointestinal comorbidities and, by modulating the gut–brain axis, potentially for aiding the treatment of depression itself.

## Author Contributions

**Xiao‐Song Xu**: writing – original draft, writing – review and editing. **Wan‐Ning Zhang**: Writing – original draft, Writing – review and editing, data curation, formal analysis. **Xu‐Rui Hao**: writing – original draft, writing – review and editing. **Xiao‐Yu Liu**: writing – original draft, writing – review and editing. **Hong‐Chen Yu**: data curation, formal analysis. **Cai‐Xia Jia**: data curation, formal analysis. **Jian‐Ming Jiang**: data curation, formal analysis. **De‐Zhi Kong**: data curation, formal analysis. **Yan‐Ru Cai**: resources, methodology, validation.

## Funding

This work was supported by the Science and Technology Project of the National Administration of Traditional Chinese Medicine (Grant No. GZY‐KJS‐2023‐025); the Hebei Provincial Central Guidance Local Science and Technology Special Project (Grant No. 246Z7708G); the Natural Science Foundation of Hebei Province (Grant Nos. H2023423001, H2023423003); theScientific Research Plan Projects of Hebei Provincial Administration of Traditional Chinese Medicine (Grant Nos. 2022032, 2024010, 2026240); the Medical Science Research Project of Hebei (Grant No. 20250859), Hebei Institute of Traditional Chinese Medicine Pharmaceutical Preparation Industry Technology (Grant Nos.YJY2024012, YJY2024015); and the Hebei Provincial Government‐Funded Clinical Medicine Excellent Talent Project (Grant No.ZF2024171).

## Ethics Statement

All experimental procedures were approved by the IACUC of the Hebei Provincial Hospital of Traditional Chinese Medicine (No. IACUC‐HPHCM‐2025009).

## Conflicts of Interest

The authors declare no conflicts of interest.

## Supporting information



Figure S1 Differences in total cell recovery between the CON(A) and M(B) groups.
Figure S2 Differentially expressed genes between CON group and M group.
Figure S3 Proportion of each cell type in stromal cell cluster.

## Data Availability

The data that support the findings of this study are not openly available but can be obtained from the corresponding author upon reasonable request.
