# Supplementary material for: Single‐Cell RNA Sequencing Reveals Disrupted Stromal–Epithelial Crosstalk Impairs Gut Barrier in Depression
Source: Brain Behav. 2026 Jul 28;16(8):e71627. doi: 10.1002/brb3.71627 (PMC13411299; doi:10.1002/brb3.71627)

Figure S1 Differences in total cell recovery between the CON(A) and M(B) groups.


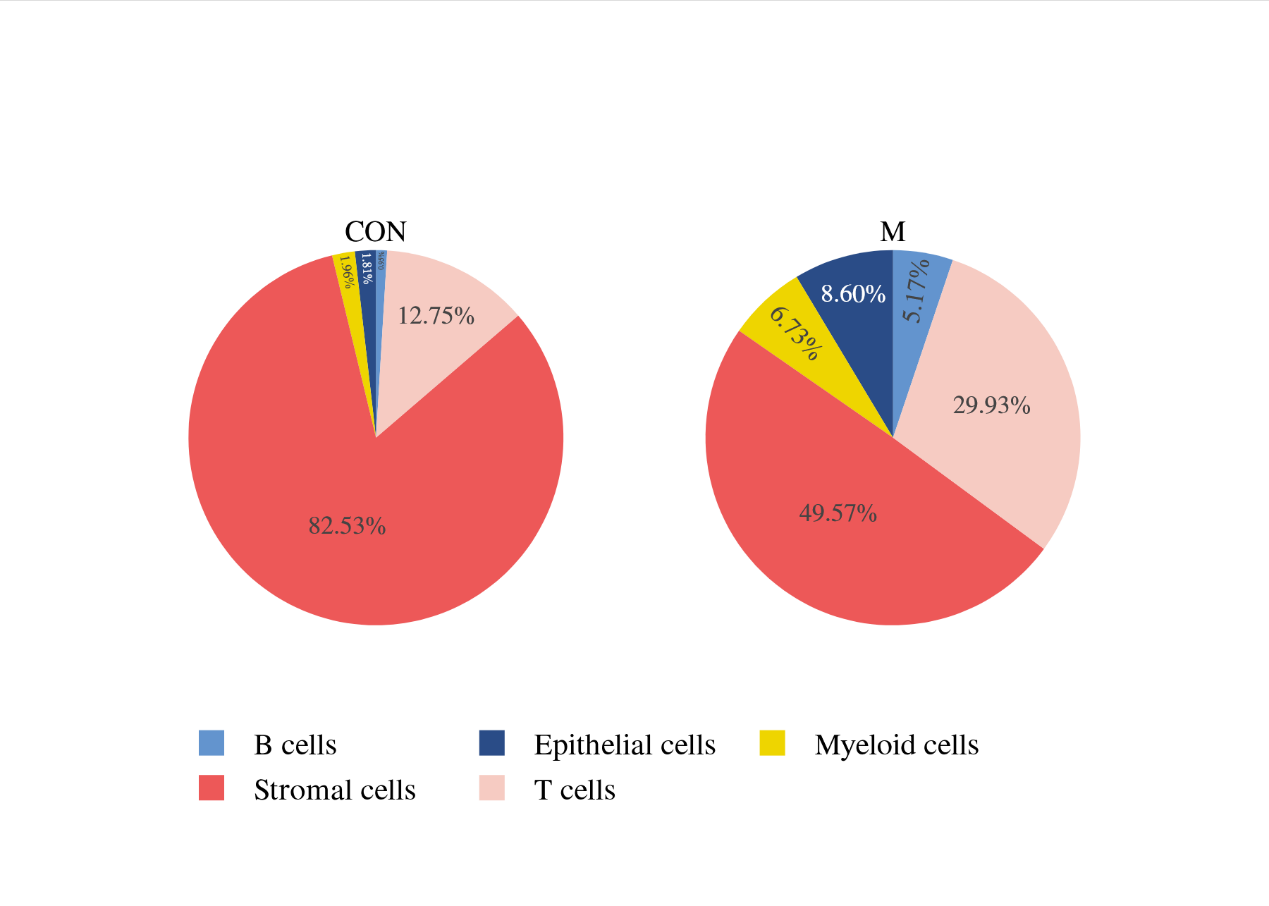


Figure S2 Differentially expressed genes between CON group and M group.


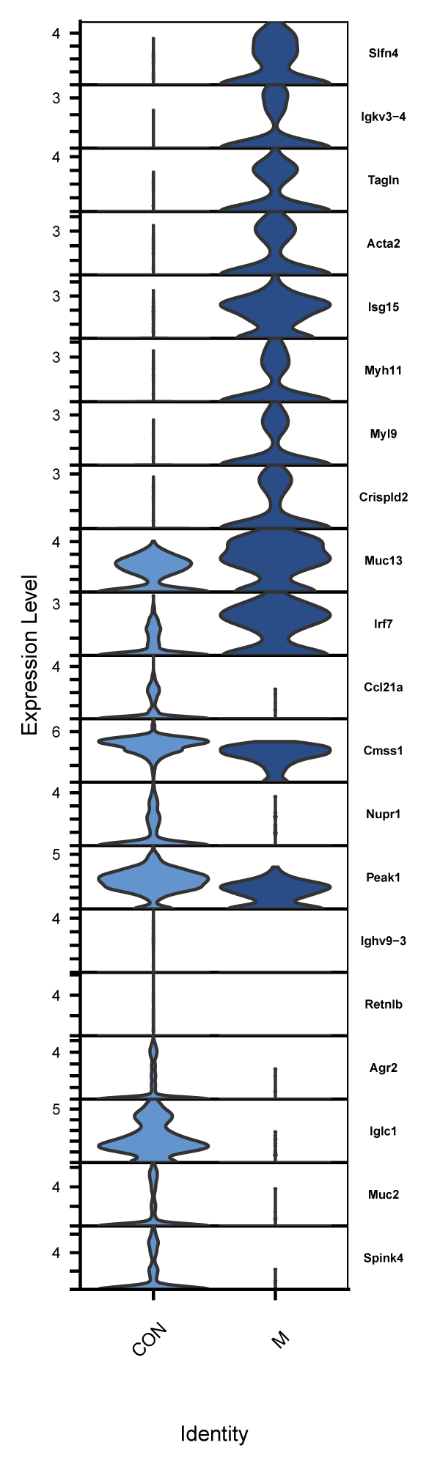


Figure S3 Differentially expressed genes of Stromal cells between CON group and M group.


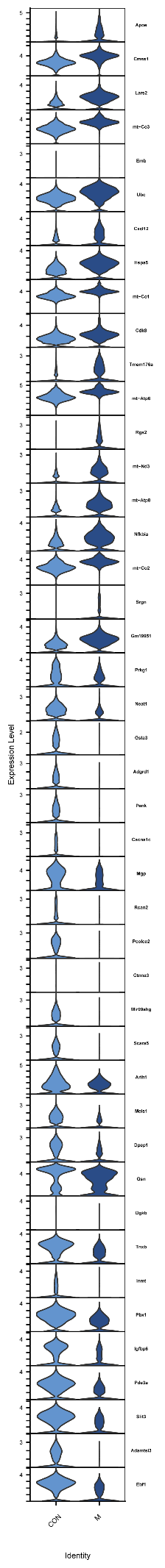


Figure S4 Proportion of each cell type in stromal cell cluster.


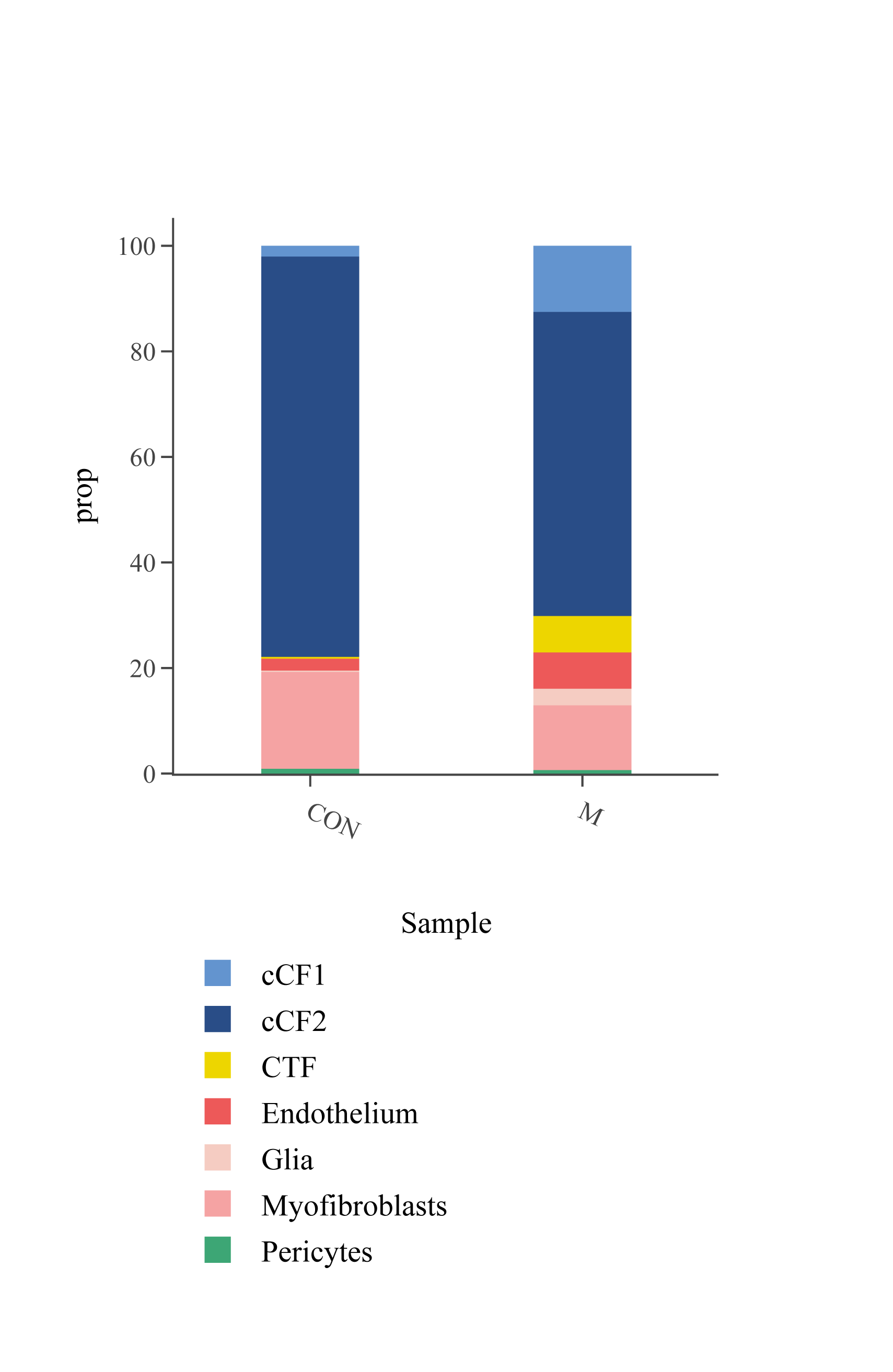

Supplement: Supplementary file 1 — Figure S1 Differences in total cell recovery between the CON(A) and M(B) groups. Figure S2 Differentially expressed genes between CON group and M group. Figure S3 Proportion of each cell type in stromal cell cluster. [file BRB3-16-e71627-s001.docx]
